# Supplementary material for: Laryngeal Cancer Cells Metabolize 25-Hydroxyvitamin D3 and Respond to 24R,25-dihydroxyvitamin D3 via a Mechanism Dependent on Estrogen Receptor Levels
Source: Cancers (Basel). 2024 Apr 24;16(9):1635. doi: 10.3390/cancers16091635 (PMC11083835; doi:10.3390/cancers16091635)
Supplement: Supplementary file 1 [file cancers-16-01635-s001.zip › cancers-2955751-supplementary.pdf]

Supplementary Methods

Supplemental Table S1: Gene specific primers.

GENE	FORWARD	REVERSE
**GAPDH**	GCTCTCCAGAACATCATCC	TGCTTCACCACCTTCTTG
**Human ERα66**	TGCCTGGAGTGATGTTTAAGC	ACGGGAGCAAGTGCAGTC
**Human ERα66/46**	TGCGTCGCCTCTAACCTCG	TCCCAGATGCTTTGGTGTGG
**Human ERα36**	TCCTCGTGTCTAAAGCCTCTG	AAAATGTCCCCACGTCCACA
**Human ESR2**	CCTCCTATGTAGACAGCCACCA	TGGCGCAACGGTTCCCACTAA
**Human GPR30**	TTCAGCAGTGCCGTGTAGA	GTGTGCAGCTCCCGAGTC
**Human VDR**	CTGCTTGTCAAAAGGCGGC	ACCCAAAGGCTTCTGGTCC
**Human BAX**	GACGAACTGGACAGTAACATGG	AAAGTAGAAAAGGGCGACAACC
**Human BCL2**	Hs_BCL2-1-SG QuantiTect Primer #249900	
**Human CYP24A1**	GACATCCAGGCCACAGACAA	ACCACCATCTGAGGCGTATT
**Human CYP27B1**	AGAGTTGCTATTGGCGGGAG	AGAACAGTGGCTGAGGGGTA
**Rat CYP24A1**	TCATCTCCCATTCGGCATCG	TCTGGTCCTTGAAGTTCGCC
**Rat CYP27B1**	CCATCGAGTCCAACTGCCTT	AGGGTCGGCCACATAAACTG

Supplemental Figure S1

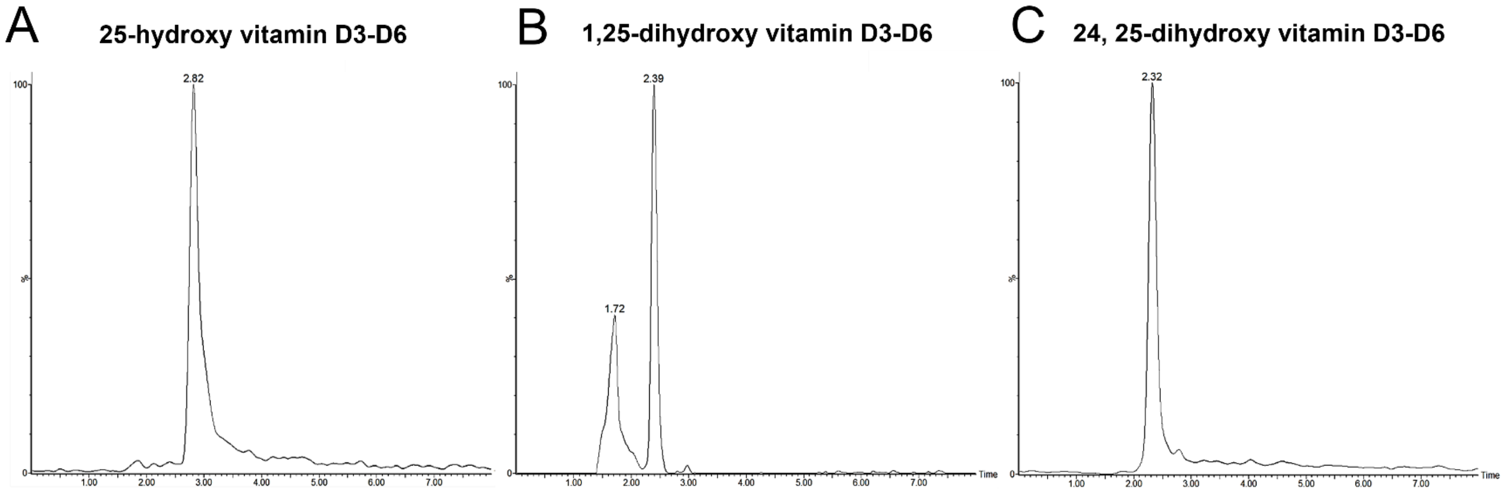

Supplemental Figure S1: Calibration curves. The calibration curve of 25-hydroxyvitamin d3-d6 (**A**), 1,25-dihydroxyvitamin d3-d6 (**B**), and 24,25-dihydroxyvitamin d3-d6 (**C**) used to create standards.

Supplemental Figure S2

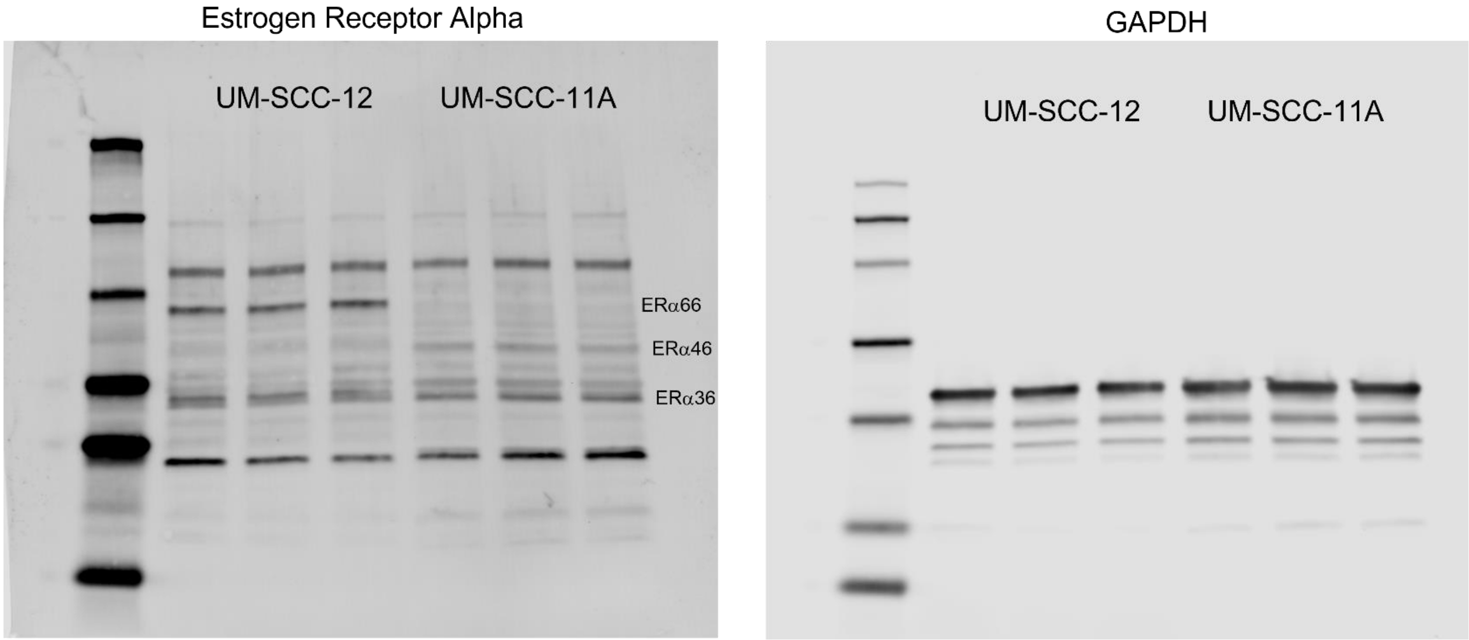

Supplemental Figure S2: Original western blots of estrogen receptor alpha protein levels in UM-SCC-12 and UM-SCC-11A cells.

Supplemental Figure S3

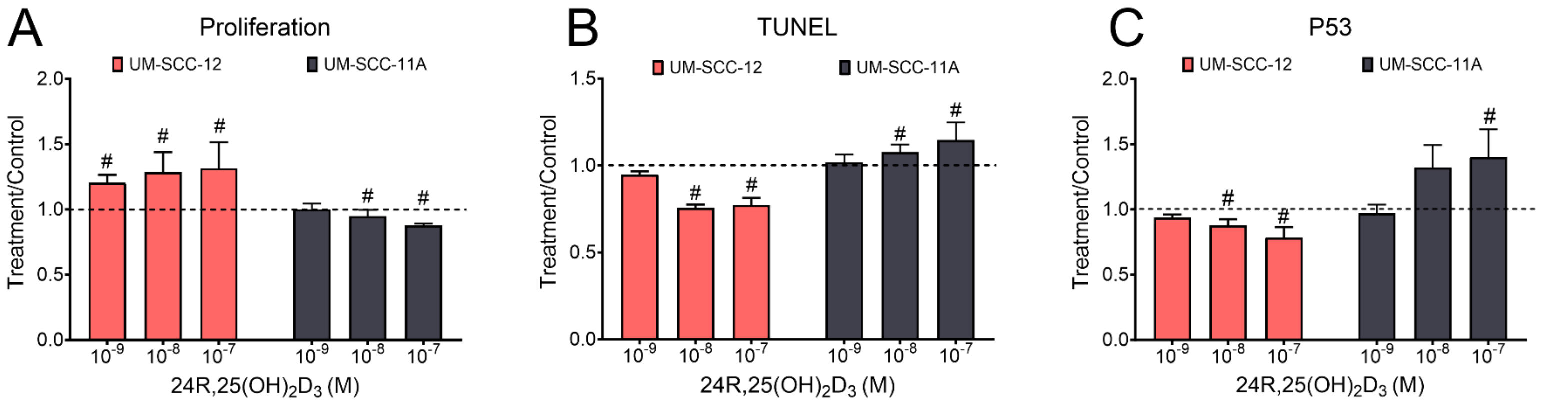

Supplemental Figure S3: Analysis of 24R,25$(OH)_2D_3$ effect on tumorigenesis. Treatment over control analysis of UM-SCC-12 and UM-SCC-11A cell proliferation after treatment with 24R,25$(OH)_2D_3$ (**A**). Treatment over control analysis of UM-SCC-12 and UM-SCC-11A cells treated with 24R,25$(OH)_2D_3$ and assessed for TUNEL (**B**). P53 analysis of UM-SCC-12 and UM-SCC-11A cells after treatment with 24R,25$(OH)_2D_3$ (**C**). Data are presented as the mean ± standard error of 3 independent experiments. Groups labeled with a "#" are statistically different compared to the vehicle control by Wilcoxon matched-pairs signed rank test with p-values ≤0.05 determined as significant.
